# Deep Learning for Semantic Segmentation of Defects in Advanced STEM Images of Steels

**DOI:** 10.1038/s41598-019-49105-0

**Published:** 2019-09-04

**Authors:** Graham Roberts, Simon Y. Haile, Rajat Sainju, Danny J. Edwards, Brian Hutchinson, Yuanyuan Zhu

**Affiliations:** 1 Nuclear Sciences Division, Pacific Northwest National Laboratory, Richland, WA 99352 USA; 2 Computer Science Department, Western Washington University, Bellingham, WA 98225 USA; 3 Department of Materials Science and Engineering, Institute of Materials Science, University of Connecticut, Storrs, CT 06269 USA; 4 Computing and Analytics Division, Pacific Northwest National Laboratory, Richland, WA 99352 USA

**Keywords:** Materials science, Structural materials, Characterization and analytical techniques

## Abstract

Crystalline materials exhibit long-range ordered lattice unit, within which resides nonperiodic structural features called defects. These crystallographic defects play a vital role in determining the physical and mechanical properties of a wide range of material systems. While computer vision has demonstrated success in recognizing feature patterns in images with well-defined contrast, automated identification of nanometer scale crystallographic defects in electron micrographs governed by complex contrast mechanisms is still a challenging task. Here, building upon an advanced defect imaging mode that offers high feature clarity, we introduce *DefectSegNet* - a new convolutional neural network (CNN) architecture that performs semantic segmentation of three common crystallographic defects in structural alloys: dislocation lines, precipitates and voids. Results from supervised training on a small set of high-quality defect images of steels show high pixel-wise accuracy across all three types of defects: 91.60 ± 1.77% on dislocations, 93.39 ± 1.00% on precipitates, and 98.85 ± 0.56% on voids. We discuss the sources of uncertainties in CNN prediction and the training data in terms of feature density, representation and homogeneity and their effects on deep learning performance. Further defect quantification using *DefectSegNet* prediction outperforms human expert average, presenting a promising new workflow for fast and statistically meaningful quantification of materials defects.

## Introduction

Crystallographic defects are critical to the properties of materials. The physical and mechanical properties of metallic materials, in particular, are controlled by crystallographic defects which in turn can be modified through proper processing routes and by service conditions^1,2^. Thus, defect analysis of structural metals and alloys is routinely carried out in metallurgy^3^ and in materials degradation studies^4,5^. Transmission electron microscopy (TEM) is one of the most important standard tools for defect characterization. Besides being capable of hosting various analytical and diffraction techniques (e.g. energy dispersive X-ray spectroscopy and precession electron diffraction)^6^, TEM imaging alone offers direct observations of a variety of property-determining defects including grain boundaries, dislocations, stacking faults, precipitates, voids, *etc*. Specifically, well-established TEM diffraction contrast theory not only offers the determination of Burger’s vector for individual dislocation lines^7^, but also provides unique insights into dislocations’ distributions and their spatial relationships with other defects that are critical for the prediction of, for example, barrier hardening effects^8^. However, the current practice of identifying defects in TEM images and deriving metrics such as dislocation density and precipitates/voids diameter remains largely in the purview of human analysis. The lack of automated defect analysis techniques for statistically meaningful quantification for various types of crystallographic defects is causing an increasingly large bottleneck for rational alloy design^9,10^.

The first and most important step of automating defect analysis is perceptual defect identification. In terms of digital image processing, semantic segmentation best emulates human recognition of defect features – it identifies the crystallographic defects and where they are located in a TEM micrograph. Early attempts were based mainly on traditional image segmentation utilizing low-level (non-specialized) cues such as pixel intensity, texture, edges, *etc*.^11^. Without involving high-level (contextual) image features, this approach is applicable mainly to simple images with sparse defects^12^. In recent years, semantic segmentation based on convolutional neural networks (CNNs) has demonstrated substantial advantages over the traditional image segmentation^13,14^, and has been successfully applied to many visual tasks such as sensing for autonomous vehicles^15^ and cell segmentation^16^. However, most reported machine learning applications in the materials science domain (excluding bio-materials), have so far only addressed the arguably easier computer vision task of image classification, i.e., classifying an entire image as one microstructure category (for example^17–20^). Semantic segmentation, able to predict both feature class and location in structural alloys, has been largely limited to large-scale phases and microstructure constituents^21,22^, or to a single type of defect^23^.

**Figure 1:**
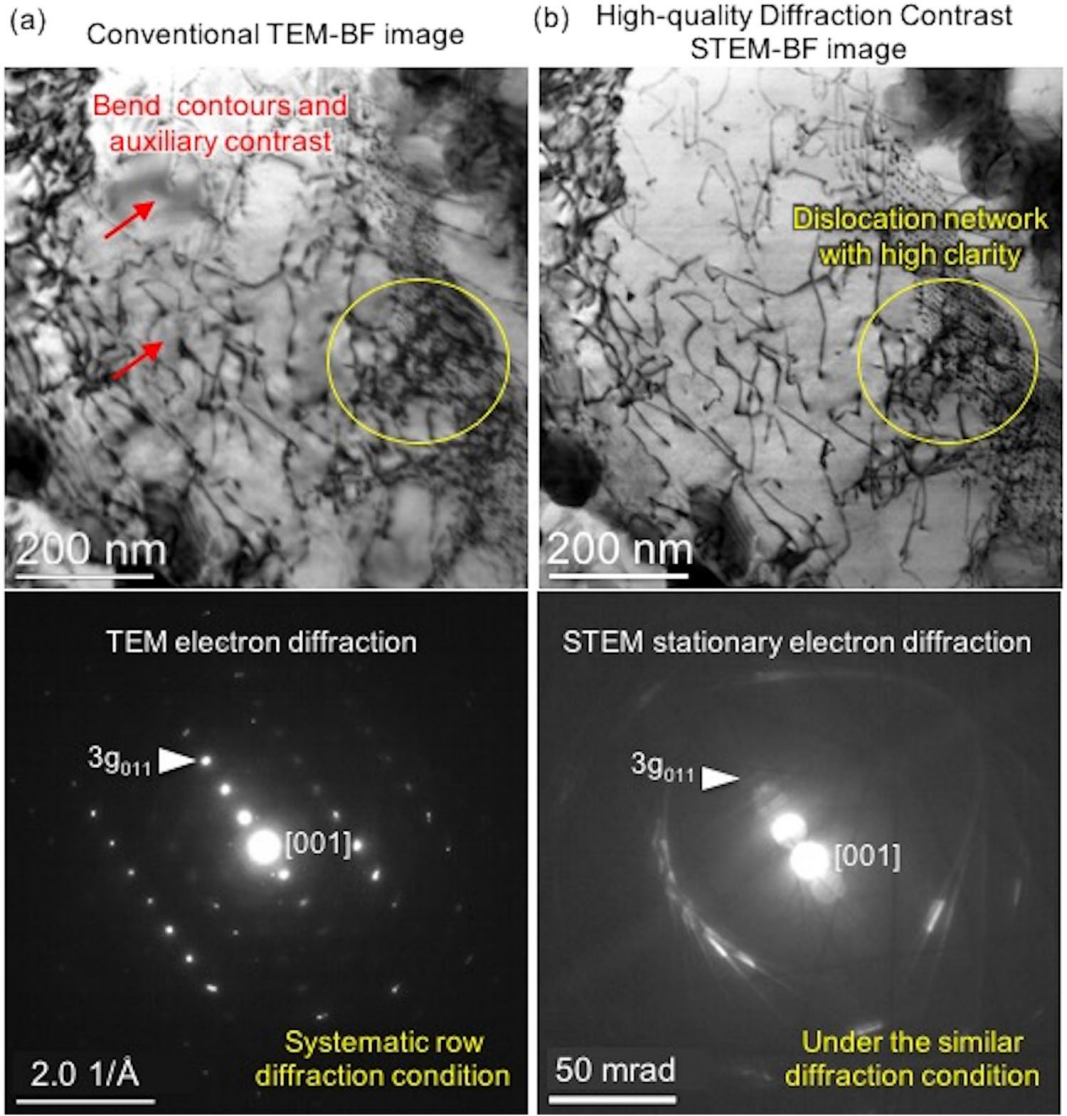
Improved clarity of dislocation images using diffraction contrast imaging scanning transmission electron microscopy (DCI STEM) in comparison with conventional bright-field (BF) TEM. (**a**) Conventional TEM-BF image of line dislocations (network) in a pristine HT-9 martensitic steel under the standard systematic row diffraction condition. Red arrows point to severe bend contour and auxiliary contrast commonly observed obscuring defect contrast. (**b**) DCI STEM image of the same field of view under a similar diffraction condition. Yellow circles highlight the sharp defect contrast in DCI STEM image.

**Figure 2:**
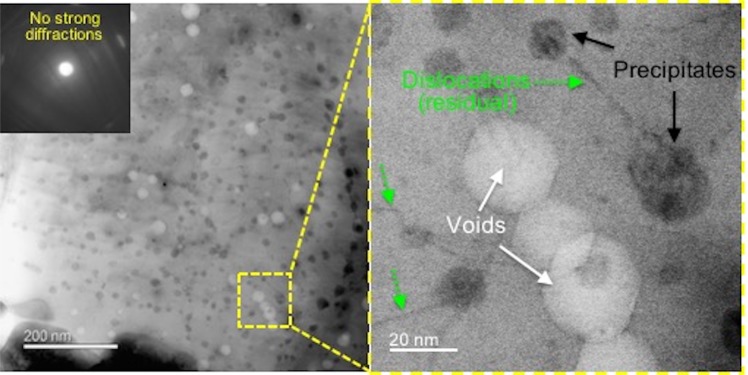
Versatile DCI STEM imaging offers high-quality defect imaging for two other crystallographic defects precipitates and voids. Off-diffraction DCI STEM image of the same HT-9 martensitic steel after introducing precipitates and voids by neutron irradiation. Enlarged region presents nanometer scale defects of precipitates in dark contrast, voids in bright contrast, accompanied by residual line dislocations.

One of the main reasons why defect semantic segmentation in TEM micrographs is a challenging deep learning task can be attributed to the nature of the images. Unlike everyday photographs, the interpretation of image contrast in TEM micrographs is often not straightforward; multiple contrast mechanisms may contribute to the observation of the defect features. A good practice is to promote one dominant contrast condition. A typical example is high-angle annular dark-field scanning transmission electron microscopy (HAADF STEM) that promotes well behaved monotonic Z-contrast (Z: atomic number). Such HAADF STEM micrographs and simulated high-resolution TEM micrographs (for precise contrast control) were employed in developing deep learning models for the recognition of atomic defects in functional nanomaterials^24,25^. However, it is a more complicated case for the diffraction contrast in imaging crystallographic defects. Conventional TEM bright-field diffraction contrast, although theoretically well defined, is known to be sensitive to practical TEM foil conditions (e.g. bending, thickness, *etc*.) and other auxiliary strain fields^26,27^. As shown in Fig. 1a, under a preferred systematic row diffraction condition, the conventional TEM imaging mode presents obvious intensity variations (e.g. bend contours) that lead to inconsistent and obscure defect contrast. These practical TEM foil conditions coupled with undesired artifacts introduce ambiguity in the ground truth labeling, and fundamentally hamper the supervised deep CNN semantic segmentation training since the fidelity of the ground truth label affects the best achievable accuracy. Moreover, because the image artifacts are dependent on local sample strain, they also give rise to a disparity in defect contrast of the same nature, posing a greater demand on feature representation and labor-intense pixel-wise labeling. Here, we aim at resolving this image-induced challenge by optimizing the image quality. In previous work, we established an experimental protocol for a diffraction contrast imaging scanning transmission electron microscopy (DCI STEM) technique and tailored it specifically for imaging defects in popular iron-based structural alloys^28^. As illustrated in Fig. 1, compared to the conventional TEM imaging mode, this new DCI STEM provided defect images of complex dislocation network with high clarity, largely free of bend contours and other image artifacts. Meanwhile, in Fig. 2, by slightly adjusting the sample tilt and suppressing strong diffractions, DCI STEM also offers almost monotonic contrast for the imaging of two other important defects – precipitates and voids. These defect images with a high clarity pave the way for the development of CNN-based defect semantic segmentation.

In this paper, we present *DefectSegNet*, a novel hybrid CNN algorithm for robust and automated semantic segmentation of three crystallographic defects, including line dislocations, precipitates and voids, that are commonly observed in structural metals and alloys. For semantic segmentation of other defects such as grain boundaries, please refer to^29^. The *DefectSegNet* was trained on a small set of high-quality DCI STEM defect images obtained from HT-9 martensitic steels (Figs 1 and 2). The performance of the resulting model for each defect was assessed quantitatively by standard semantic segmentation evaluation metrics, and the resulting defect density and size measurement was compared to that of from a group of human experts. We find that deep learning methods show a great promise towards fast, accurate and reproducible feature semantic segmentation for quantitative defect analysis.

## Methods

### Diffraction contrast imaging STEM

All defect images used for deep CNN training were acquired using the advanced DCI STEM imaging mode providing high-quality input images^28^. In this work, DCI STEM imaging was performed using a JEOL ARM200CF microscope operated at 200 kV, with a convergence semi-angle of 6.2 mrad and bright-field collection angle of 9 mrad. This imaging setting was optimized previously for the HT-9 martensitic steel with a body-centered cubic (BCC) crystal structure. To balance field-of-view size and pixel resolution, a magnification of 250,000× and a 2048 × 2048 pixels image size (i.e. a pixel size of 3.2 nm/pixel) along with a dwell time of 16 µs was used to acquire all DCI STEM images. For imaging dislocations (e.g. Fig. 1), the commonly used systematic row diffraction condition was satisfied by tilting a TEM sample of pristine HT-9 steel away from [001] zone axis to approximately 1***g***_011_ on Bragg condition. The line dislocations in this BCC crystal were identified as the ½〈111〉{111} dislocation. Moreover, optimal defect contrast for precipitates and voids can be achieved by slightly tilting the TEM sample (about 2 to 4 degrees) off the systematic row diffraction condition until there are no strongly excited diffractions (Fig. 2). Here, the same HT-9 martensitic steel after neutron irradiation at 412 °C with a high-density of induced precipitates and voids defects was employed to provide good defect feature representation. For details on the DCI STEM imaging method and TEM sample preparations, one may refer to our previous study^28^.

### Image pre-processing and labeling

Prior to ground truth labeling, the DCI STEM micrographs were preprocessed including background subtraction and full variance normalization^30^ to further enhance defect contrast in regions where diffraction condition is not ideal. Each precipitate and void image (e.g. Fig. 2a), after the pre-processing, was then plotted into two images with reversed intensity. In this way, all images present bright-contrast defect features on a dark background (Fig. S1a). The ground truth labeling of the pre-processed micrographs was created by manual annotation. Lines with a width of 3 pixels were used to segment the dislocations. For voids and precipitates, after identifying the feature outline the inner region was filled evenly. As shown in Fig. S1b, all labeled features were assigned an intensity of 255, and background intensity is 0. Three researchers experienced in defect analysis worked collaboratively and cross-examined the ground truth labels over several iterations. Great care was taken throughout the labeling process to achieve, to a large extent, a pixel-level precision.

### Image augmentation

**Figure 3:**
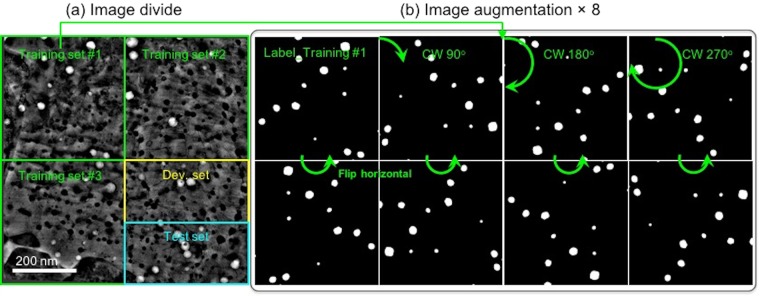
Illustration of (**a**) division and (**b**) augmentation of a pre-processed DCI STEM voids image (2048 × 2048 pixels). For a clear illustration, the label of training set #1 (1024 × 1024 pixels) was employed to show data augmentation.

To reduce the risk of overfitting^31^, a data augmentation strategy was applied to the input images and their corresponding labels. As demonstrated in Fig. 3a, a full 2048 × 2048 pixels image was divided into five regions, including three training subsets (each 1024 × 1024 pixels), and one development set and one test set (each 1024 × 512 pixels). Then, each training subset was augmented by rotation (i.e. 90°, 180°, and 270° clockwise) and by horizontal flipping each rotated image. This increases the training set size by a factor of 8, yielding new training data sets (both images and labels) that are not identical but maintain the defect features present in the images. The development sets and testing sets are not augmented. In all, two original 2048 × 2048 pixels micrograph/label sets are augmented to produce 48 1024 × 1024 pixels training image and label pairs used for the training of deep CNN models for defect semantic segmentation.

### Semantic segmentation deep CNN architecture and *DefectSegNet*

**Figure 4:**
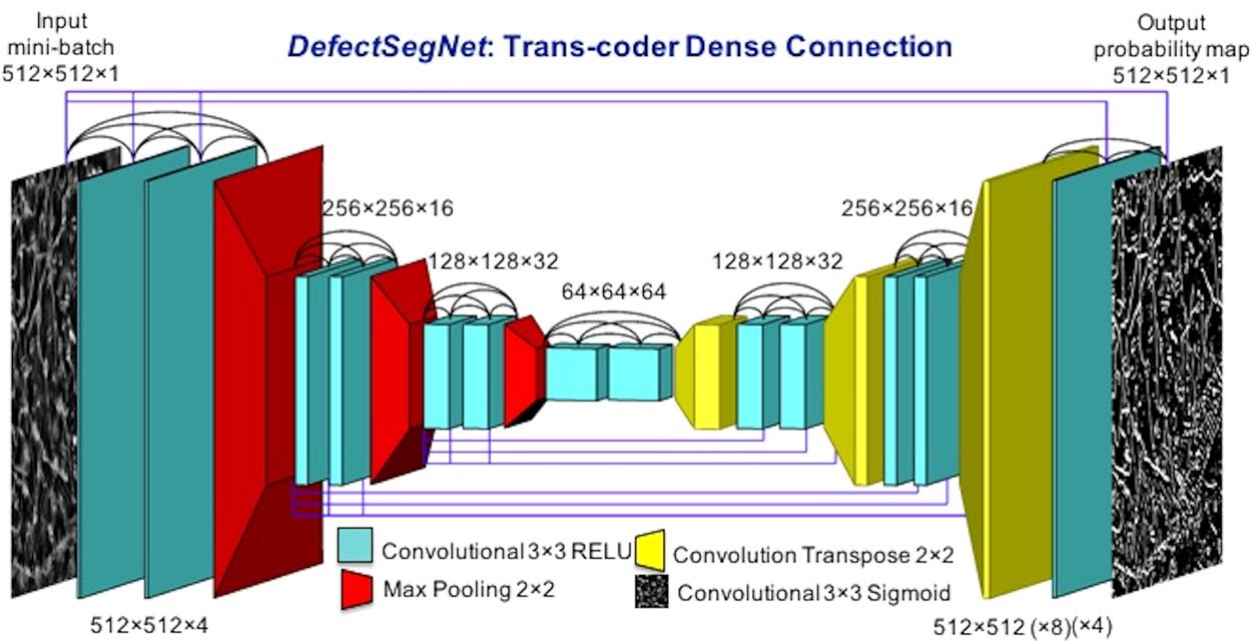
Schematic illustration of the *DefectSegNet* architecture. The final softmax layer outputs a pixel-wise classification for each defect type. Note that not only each dense block but also the feature maps with the same spatial resolution across the encoder and decoder are all connected.

Semantic image segmentation is a pixel-wise dense classification computer vision task. While the end goal of a deep image classification network is to classify an entire image (i.e. predict the class presence probability), semantic segmentation requires semantically meaningful discrimination at the pixel level^14,32^. Thus, a general semantic segmentation deep CNN architecture typically consists of two parts: an encoder functioning in a similar fashion to classification network like AlexNet^33^, VGG^34^, etc., and a decoder that projects discriminative high-level (low-resolution) features back to high-resolution space to achieve pixel-wise classification. Among the large variety of deep semantic segmentation architectures today, the biggest differences are in the design of the decoder (e.g. in the choice of up-sampling mechanism) and the design of skip connections within the network. For example, the ground-breaking Fully Convolution Networks (FCNs)^35^ utilizes bilinearly initialized interpolation for up-sampling and simple addition to fuse features from the encoder to decoder path. The U-Net^36^ which is known for effective performance in data-limited scenarios, proposed a 2 × 2 “up-convolution” path, combined with skip concatenation connections allowing the decoder to leverage relevant encoder feature maps at each stage. Recently, the DenseNet^37^ model took the design of skip connections further and introduced dense blocks within which there is an iterative concatenation of previous feature maps. In this work, we explored several hybrid deep networks for pixel-wise semantic segmentation of the three defect features. Our *DefectSegNet* was inspired by the U-Net and DenseNet and we find it offers the best performance, particularly for dislocations. The *DefectSegNet* architecture, shown in Fig. 4, consists of a total of 19 hidden layers. On the encoder side, max pooling is performed after each dense block, enabling the succeeding block to extract higher level, more contextual (and abstract) features from the defect images. For the decoder, to recover the resolution we employed the transposed convolutions, a more sophisticated operator than bilinear interpolation^35^, for up-sampling. There are equal numbers of max pooling layers and transposed convolution layers, so the output probability map has the same spatial resolution as the input image. For the design of skip connections, besides those already introduced in dense blocks, feature maps created during encoding are input to all the decoder layers of the same spatial resolution. This allows the feature maps of a certain spatial resolution to connect cross the encoder-decoder performing in a similar manner to a single dense block. The incorporation of these skip connections both within and across blocks is the primary difference between our *DefectSegNet* and the U-Net^36^ and the fully convolutional DenseNet^38^. Lastly, the final hidden layer is a 3 × 3 convolutional layer with a sigmoid activation function for classification.

### Training procedure

All of the deep learning networks were trained using TensorFlow^39^ with a batch size of 16 image patches of 512 × 512 pixels. To prevent overfitting several regularization techniques were implemented in addition to data augmentation, including L2 regularization^40^, Dropout^41^ and early stopping^42,43^. Meanwhile, IU curves of the training and development sets were monitored as a training protocol to inform possible overfitting. A learning rate (i.e. optimization step size) ranging between 0.00001 and 0.01 was tuned as a hyperparameter. For each experiment, the training was conducted for 100 passes through the training set (epochs). The learning rate was decayed each time that ten epochs without improvement was encountered. Training was terminated after the sixth learning rate decay. To compensate for class imbalance, we modified the pixel-wise cross-entropy loss function^42^, which is commonly used in segmentation tasks, by adding a tunable weight coefficient (i.e. a hyperparameter) that scales each positive pixel’s contribution to the cross-entropy loss. As training progresses, the weight coefficient is decreased, and this weighted loss function was minimized by the Adam optimizer. For each defect feature (and each architecture tested), the network was trained over a collection of random configurations of hyperparameters, and then evaluated on development sets. The top-performing models in each training experiment were then saved to warm start additional models (i.e. initializing with the weights and biases of the previously best prediction) and were further trained and then evaluated on the development sets until no performance improvement was observed. Lastly, the best model was applied to test sets. All training and evaluation for the experiments reported in this manuscript were carried out at PNNL’s Institutional Computing Cluster using NVIDIA P100 GPUs.

### Performance evaluation

In this work, we first report four evaluation metrics common for semantic segmentation tasks^21,35^. In particular, to account for the class imbalance between defects and background pixels, informative metrics besides pixel accuracy are also evaluated. To facilitate the assessment of pixel-wise dense classification in semantic image segmentation, a confusion matrix consisting of true positive (TP), true negative (TN), false positive (FP) and false negative (FN) at each pixel of prediction maps is used to introduce the evaluation metrics below,

- Pixel accuracy: the percentage of pixels correctly predicted by *DefectSegNet*.
  $${\rm{Pixel}}\,{\rm{accuracy}}=({\rm{TP}}+{\rm{TN}})/({\rm{TP}}+{\rm{FP}}+{\rm{TN}}+{\rm{FN}})$$
- Precision (positive predictive value): the fraction of pixels that are true positives (correctly predicted pixels of the targeting class) among the total positive predictions; it penalizes false positives that could lead to overestimation.
  $${\rm{Precision}}={\rm{TP}}/({\rm{TP}}+{\rm{FP}})$$
- Recall (true positive rate): the fraction of pixels that are true positives among the total class-relevant pixels; it penalizes false negatives that could cause underestimation.
  $${\rm{Recall}}={\rm{TP}}/({\rm{TP}}+{\rm{FN}})$$
- Region intersection over union (IU or IoU): the fraction of pixels that are true positives among the union of pixels that are positive predications and belong to the target class. Since both FP and FN are included in the denominator of IU, it penalizes both over and under estimations.

$${\rm{IU}}={\rm{TP}}/({\rm{TP}}+{\rm{FP}}+{\rm{FN}})$$

In addition, to assess the practical impact of the deep learning enabled defect semantic segmentation, a series of quantitative defect metrics that are directly relevant to alloy research are measured from the *DefectSegNet* predicted defect maps. These materials metrics include (1) dislocation density, (2) precipitates/voids number density and (3) precipitates/voids particle sizes (diameter) and the standard deviation of the particle diameter. Measurement methods such as the grid-intersection method for dislocation density estimation^44^ are quite standard in the metallurgy community, thus the outcomes reflect mainly how the imperfections in defect semantic segmentation translate into errors in determining these materials metrics. We carried out these standard defect quantifications in a set of dedicated MATLAB algorithms^45^ developed in-house to automate this process. In parallel, a group of six experienced human experts performed independent defect analysis on the same test images. The metrics generated by both the algorithm and the human experts were compared to the ground truth. Note that the three researchers who produced the ground truth did not participate in the manual defect quantification to ensure the integrity of the comparison.

## Results and Discussion

### *DefectSegNet* semantic segmentation of line dislocations

**Figure 5:**
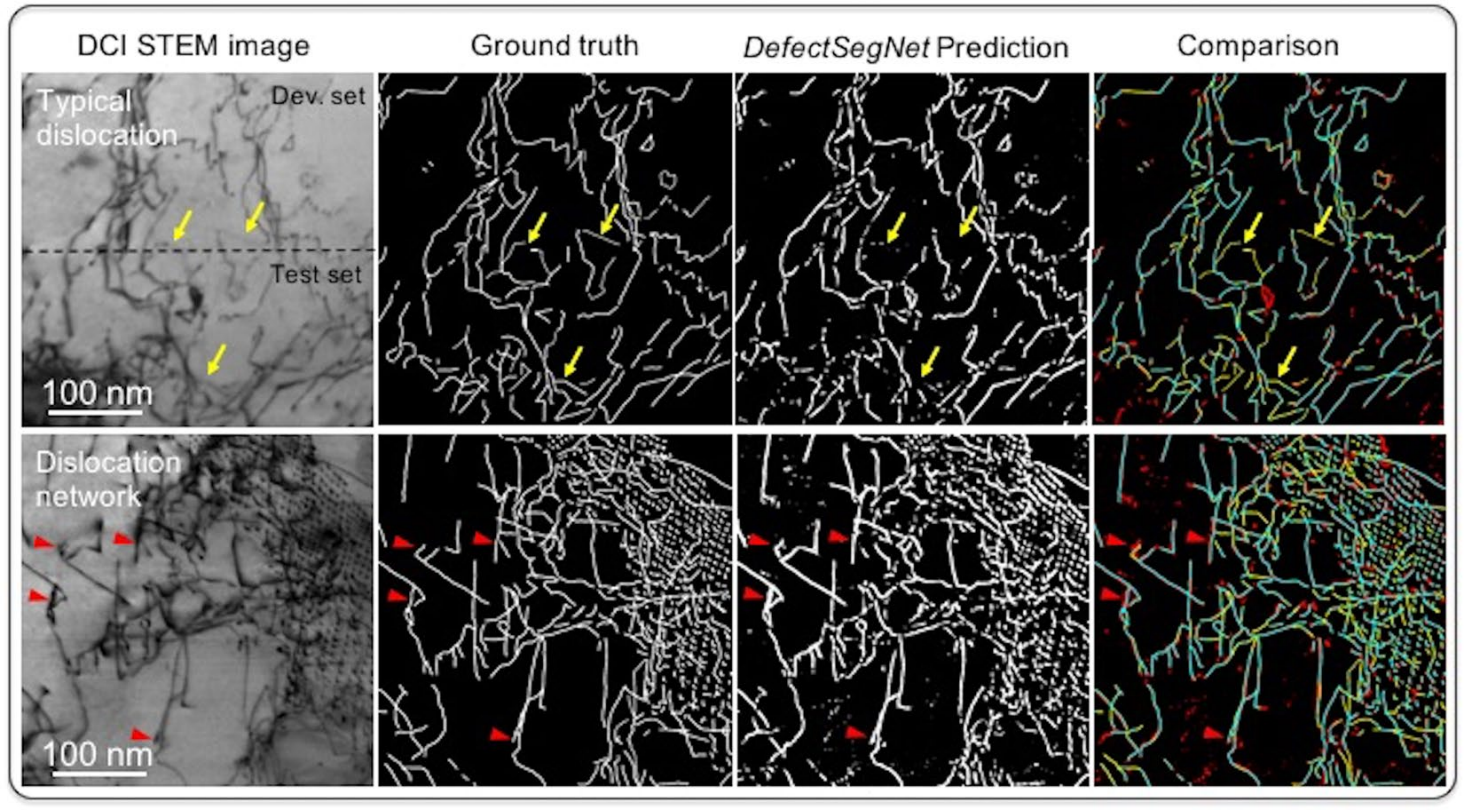
*DefectSegNet* pixel-wise semantic segmentation prediction of line dislocations using DCI STEM images. The corresponding ground truth labels, *DefectSegNet* prediction maps and the comparison maps color coded based on the confusion matrix: true positive (turquoise), true negative (black), false positive (red) and false negative (yellow) at each pixel for both development and test sets. Yellow arrows mark uncommon dislocation lines with weak contrast, and red arrows point to overestimation of FP.

Figure 5 presents the *DefectSegNet* semantic segmentation predictions for the development/validation sets and the test sets (combined as a squared 1024 × 1024 pixels image) of the first defect type: line dislocations. Comparing the ground truth label with the deep learning predicted dislocation maps (both are binary images) shows satisfactory resemblance, especially for the complex case of the dislocation network. Table 1 summarizes the semantic segmentation performance of the *DefectSegNet* on the test sets. A pixel accuracy of 91.60 ± 1.77% and an IU of 44.34 ± 0.63% was achieved for the dislocation lines. To correlate this prediction performance with the defect image characteristics, we applied a color-coded confusion matrix, i.e. TP in turquoise (defect feature), TN in black (background), FP in red and FN in yellow, at each pixel of the dislocation prediction map, providing direct visualization of the model performance. We can see that the majority of pixels in the prediction map are in black and turquoise and thus correctly classified as the background and the dislocations, respectively. In particular, striking details in the top right corner of the complex dislocation network were almost perfectly predicted by the *DefectSegNet* model. This might be attributed to the incorporation of the dense skip connections in the architecture of the *DefectSegNet*, which enable precise feature localization by directly propagating information across high-resolution feature maps. Even the early FCNs^35^ included some skip connections to preserve and reuse feature maps at different pooling stages, while the DenseNet^37^ took it further by iteratively concatenating feature maps within each dense block to aid propagation of information through the network. Considering that defect features such as line dislocations possess both distinctive location and extended features, we designed the *DefectSegNet* to leverage “dense skip connections” across the encoder and decoder (blue lines in Fig. 4). Among the several hybrid CNN models we tested so far, the *DefectSegNet* with dense skip connections offers the best semantic segmentation performance for the dislocations and for the three defects overall (Table S1). We analyzed the source of the CNN prediction uncertainties. In Fig. 5, the red FP and yellow FN pixels in the comparison maps suggest that the uncertainties are probably related to feature representation and to the protocol of ground truth labeling. As indicated by the yellow arrows, several dislocation lines exhibiting a relatively weak contrast were missed (FN) by the model. The occasional presence of these dislocations with weak contrast is due to the fact that diffraction contrast is sensitive to local lattice strain, which sometimes leads to unsatisfying the dislocation contrast. These underrepresented input patterns can then give rise to missed predictions and affect the corresponding recall (and the IU), especially when training data is small. Although this problem can usually be mitigated by increasing the training data set, the cost of additional ground truth labeling is often high in semantic segmentation; this is particularly true for our microscopy data. Here, we further assessed the situation by evaluating the material metric related to dislocations, i.e. dislocation density, in the third section below. Moreover, the model also produces some false positives (in red). Some can be attributed to background noise (and thus are legitimate false alarms), while others reflect a deficiency in ground truth annotations. As marked by the red arrows in Fig. 5, the red FP pixels surrounding the dislocation lines are in fact due to that the fixed width adopted for dislocation line label (3 pixels) is too narrow to capture the full defect. Despite the fact that this leads to an increased FP rate (and lower precision) in semantic segmentation evaluation (Table 1), the ground truth was kept as it is since the width of a dislocation line does not affect the final dislocation density measurement.

**Table 1:** Semantic segmentation performance of the *DefectSegNet* for the crystallographic defects in steel.

*DefectSegNet* Performance	Pixel accuracy	Precision	Recall	IU
Dislocations	91.60 ± 1.77%	55.37 ± 2.22%	69.10 ± 1.93%	44.34 ± 0.63%
Precipitates	93.39 ± 1.00%	72.06 ± 4.44%	78.38 ± 2.05%	59.85 ± 2.07%
Voids	98.85 ± 0.56%	89.17 ± 1.28%	90.17 ± 5.84%	81.19 ± 3.68%
**Defect Overall**	**94.61** ± **1.13%**	**72.12** ± **2.73%**	**79.22** ± **3.27%**	**61.79** ± **2.13%**

The number reported here are averaged metrics with standard deviation over test sets.

### *DefectSegNet* semantic segmentation of precipitates and voids

**Figure 6:**
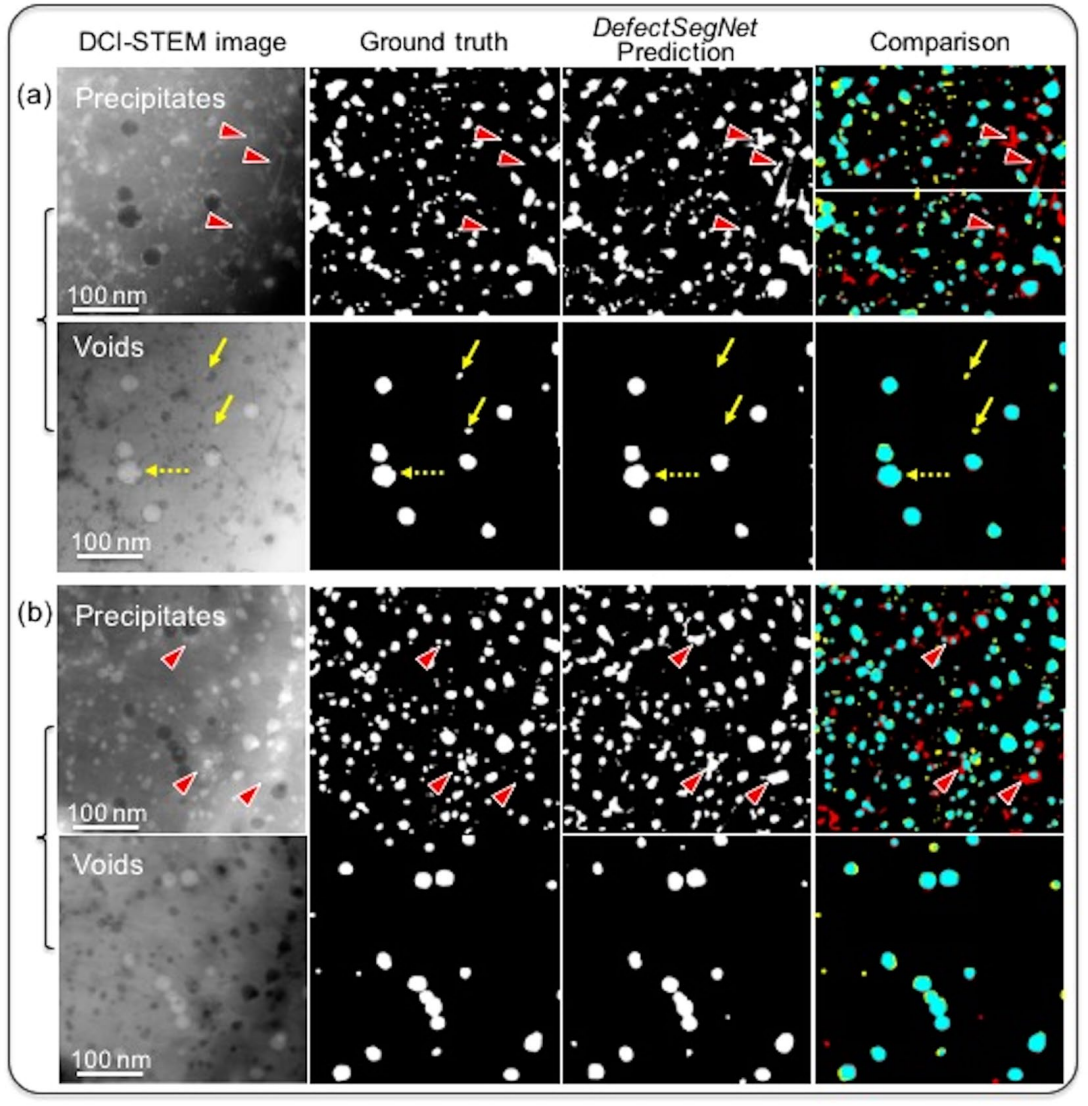
*DefectSegNet* pixel-wise semantic segmentation prediction of precipitates and voids using pairs of DCI STEM images. (**a**) Set #1 and (**b**) #2 precipitates and voids pairs and their corresponding ground truth, *DefectSegNet* prediction maps and comparison maps with the same confusion matrix color coding as Fig. 5. Similarly, the development/validation and the test sets are combined as a squared 1024 × 1024 pixels image for better illustration. Red arrows mark the source of false positives for precipitate prediction, and yellow arrows point to overlapped voids with precipitates.

Compared to the evaluation metrics of the dislocations (Table 1), both precipitates and voids present higher pixel accuracies with a particular high accuracy of 98.85 ± 0.56% for voids prediction. A more dramatic improvement is observed in the evaluation of precision, recall and IU for these two defects. In particular, the IU of precipitates is 59.85 ± 2.07%, and 81.19 ± 3.68% is achieved for voids. Here, we first investigate false positive errors. The *DefectSegNet* semantic segmentation prediction and comparison maps of the precipitates and voids are shown in Fig. 6. Marked by red arrows, two sources of false positive in precipitate prediction are identified, (1) local residual dislocation contrast (Fig. 6a), and (2) occasional lattice strain induced image contrast dilates the size of precipitates (Fig. 6b). These false alarms lead to the precision (72.06 ± 4.44%) being lower than the recall (78.38 ± 2.05%) for the semantic segmentation of precipitates. In contrast, the precision and recall for voids are similar (~90%). Noticeable false negatives (yellow arrows in Fig. 6) appear to be related to the precipitates that overlap with voids (opposite contrast canceled out). In all, except for few uncommon features that induce false predictions, the *DefectSegNet* has demonstrated an excellent performance in semantic segmentation of particle-like defects with an average IU of ~70%. The current *DefectSegNet* was trained over a limited number of labeled DCI STEM images, but it achieved quite promising semantic segmentation performance with an overall accuracy of ~95% and an overall IU of ~62% for the three defects. In computer vision, the size of a training set, which is usually judged by the number of images, is known to be an important factor for model performance. This is particularly true for image classification. For semantic segmentation tasks, we argue that the training data in data-driven learning are the features rather than the images. The performance of semantic segmentation models depends highly on the density and homogeneity of the features to be identified in the input images. By choosing an HT-9 sample with a high-density defect features, despite being limited to only two training images for precipitates and voids prediction, our training set contains 823 precipitates and 110 voids. Moreover, we also noticed that unlike certain features exhibiting different shapes and with a complex combination of contrast^23^, both precipitates and voids have a rather monotonic contrast and uniform feature representation. This makes our training set of several hundred repeating features sufficient supervision for the *DefectSegNet* to achieve strong generalization. Furthermore, as discussed above, many of the incorrect predictions can be attributed to uncommon features such as the dislocations with weak contrast or the lattice strain induced additional contrast. In this work, the adoption of the advanced DCI STEM for defect imaging that largely eliminates bend contours and other auxiliary contrast, is a valuable step in reducing the abnormalities and improving feature homogeneity in training sets. Thus, for dense classification tasks like semantic segmentation, in addition to the size of training data, the representation and quality of the features play an important role in model performance.

### Defect quantification metrics and comparison with human experts

**Figure 7:**
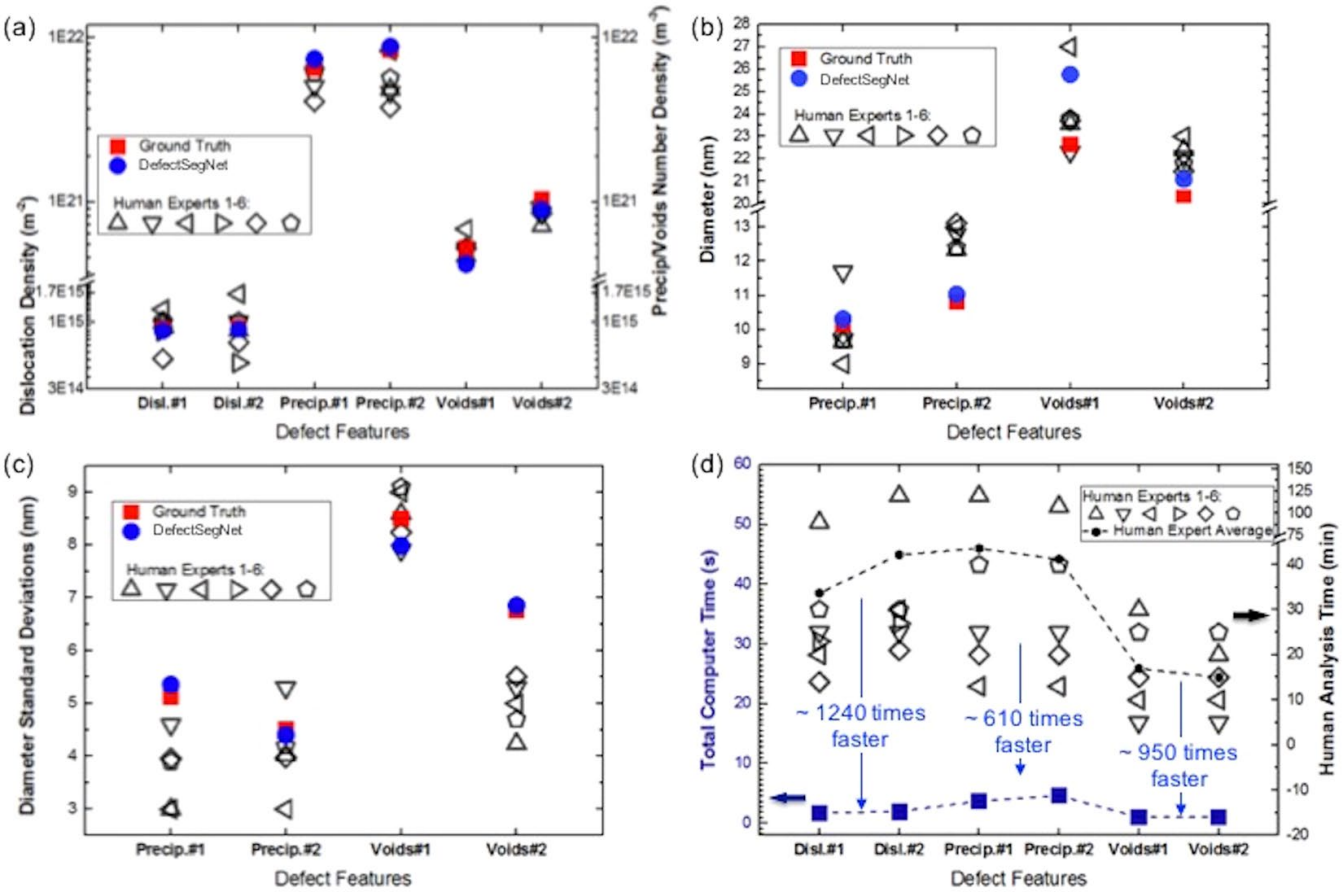
Comparison of materials evaluation metrics for defect quantification performed by computer and by human experts. Materials metrics include (**a**) dislocation density, and precipitates and voids number density, (b) the diameter and (**c**) diameter standard deviation of precipitates and voids. (**d**) The time spend for computer and human experts to quantify these defects. The defect set number is corresponding to the image sequence in Figs 5 and 6.

How the above semantic segmentation evaluations translate into the more practical materials evaluations is discussed in this section. Figure 7 presents the plots of materials evaluation metrics for defect quantification performed by computers and by human experts. Among all categories of the defect quantifications, including dislocation density, number density, diameter, and diameter standard deviation of precipitates and voids, except for one set of data, the computer-based method provides an overall more accurate result. To quantitatively evaluate the degree of accuracy, the absolute percent errors were calculated and summarized in Tables 2, 3. Taking dislocation density for example, for set #1 (the typical dislocation in Fig. 5) the ground truth density is 8.91 × 10^14^ m^−2^. The *DefectSegNet* gives a result of 8.58 × 10^14^ m^−2^ with a small error of ~4%. It’s interesting to see that while the occasional dislocation lines with weak contrast affect the recall (and IU), they do not seem to induce a large error in the density quantification. In contrast, a density value between 5.20 × 10^14^ to 1.26 × 10^15^ m^−2^ with an average error of ~20% was produced by six human experts. A similar gap in percent error can also be found in the quantification of precipitates number density and size. In particular, due to that the precipitates are high in density and small in size, the human quantification of precipitate number density becomes less reliable, with an average error of ~45%, while the *DefectSegNet* limits the error to ~10% on average. A very small error of only ~2% for the *DefectSegNet* was achieved in determining the precipitates size; whereas, average human error was around ~13%. One case where humans performed better was during the analysis set #1 voids. As discussed above (yellow arrows in Fig. 6a), due to that the two small voids overlap with precipitates, the resulting abnormally low void contrast leads to missed predictions. Although it only leads to mild reduction in recall and IU (since a small number of pixels are involved), in this case of sparse voids in the field of view, missing two counts results in an error of 20% in number density, and of 14% in diameter quantification. It is recommended to carry out a quick manual check after the automated semantic segmentation to catch such missed voids. Lastly, when comparing the time efficiency of the quantification methods, as shown in Table 4 and Fig. 7d, the computer-assisted analysis performs better by a large margin. For the defect quantification that typically takes at least half an hour even for an expert, the *DefectSegNet* and associated MATLAB algorithms can produce results in a more reproducible and reliable manner in a few seconds.

**Table 2:** Defect quantification results of dislocation density and the number density of precipitates and voids performed by computer and by human experts.

Defect quantification	Density (m^−2^)		Number density (m^−3^)			
	Dislocations #1	Dislocations #2	Precipitates #1	Precipitates #2	Voids #1	Voids #2
Ground Truth	8.91E + 14	9.19E + 14	6.56E + 21	8.42E + 21	5.25E + 20	1.04E + 21
*DefectSegNet* Prediction	8.58E + 14	8.78E + 14	7.46E + 21	8.87E + 21	4.20E + 20	9.00E + 20
**Machine Percent Error**	**0**.**04**	**0**.**05**	**0**.**14**	**0**.**05**	***0***.***20***	**0**.**13**
Human Expert 1	9.96E + 14	8.61E + 14	7.04E + 21	4.86E + 21	4.73E + 20	7.20E + 20
Human Expert 2	9.50E + 14	1.01E + 15	5.09E + 21	4.68E + 21	5.25E + 20	8.55E + 20
Human Expert 3	1.26E + 15	1.66E + 15	2.08E + 22	8.36E + 21	6.88E + 20	9.90E + 20
Human Expert 4	8.45E + 14	4.81E + 14	\	\	\	\
Human Expert 5	5.20E + 14	6.92E + 14	4.10E + 21	3.78E + 21	5.25E + 20	8.56E + 20
Human Expert 6	1.03E + 15	1.02E + 15	6.10E + 21	5.70E + 21	5.30E + 20	9.00E + 20
**Avg**. **Human Percent Error**	**0**.**20**	**0**.**30**	**0**.**58**	**0**.**35**	**0**.**08**	**0**.**17**

**Table 3:** Defect quantification results of precipitates and voids diameter and diameter standard deviation performed by computer and by human experts.

Defect quantification	Precipitates #1		Precipitates #2		Voids #1		Voids #2	
	Diameter (nm)	StDev. (nm)	Diameter (nm)	StDev. (nm)	Diameter (nm)	StDev. (nm)	Diameter (nm)	StDev. (nm)
Ground Truth	10.17	5.12	10.83	4.51	22.64	8.50	20.26	6.76
*DefectSegNet Prediction*	10.32	5.36	11.04	4.41	25.77	7.99	21.09	6.86
**Machine Percent Error**	**0**.**01**	**0**.**05**	**0**.**02**	**0**.**02**	***0***.***14***	**0**.**06**	**0**.**04**	0.01
Human Expert 1	9.65	3.00	12.35	4.03	23.56	8.60	22.41	4.24
Human Expert 2	11.70	4.60	12.80	5.30	22.30	7.90	22.10	5.30
Human Expert 3	9.00	3.00	13.00	3.00	27.00	9.00	23.00	5.00
Human Expert 4	\	\	\	\	\	\	\	\
Human Expert 5	9.86	3.96	13.12	3.98	23.71	8.24	21.45	5.51
Human Expert 6	9.70	3.90	12.40	4.20	23.80	9.10	21.80	4.70
**Avg**. **Human Percent Error**	**0**.**08**	**0**.**28**	**0**.**18**	**0**.**16**	**0**.**07**	**0**.**05**	**0**.**09**	0.27

**Table 4:** Time spend on quantitative defect analysis for the three defects by computer and by human experts.

Time Spend	Dislocations #1	Dislocations #2	Precipitates #1	Precipitates #2	Voids #1	Voids #2
*DefectSegNet* Segmentation (s)	0.025	0.027	0.027	0.026	0.030	0.025
MATLAB Defect Quantification (s)	1.69	1.92	3.73	4.66	0.97	1.01
**Total Computer Time (s)**	**1**.**71**	**1**.**95**	**3**.**76**	**4**.**69**	**1**.**00**	**1**.**03**
Human Expert 1 (min)	90.0	120.0	120.0	108.0	30.0	20.0
Human Expert 2 (min)	25.0	25.0	25.0	25.0	5.0	5.0
Human Expert 3 (min)	20.0	30.0	13.0	13.0	10.0	10.0
Human Expert 4 (min)	23.0	27.0	\	\	\	\
Human Expert 5 (min)	14.0	21.0	20.0	20.0	15.0	15.0
Human Expert 6 (min)	30.0	30.0	40.0	40.0	25.0	25.0
**Human Expert Average (min)**	**33**.**7**	**42**.**2**	**43**.**6**	**41**.**2**	**17**.**0**	**15**.**0**

### Concluding remarks

We demonstrate the feasibility of automated identification of common crystallographic defects in steels using deep learning semantic segmentation, based on high-quality microscopy data. In particular, the *DefectSegNet* – a new hybrid CNN architecture with skip connections within and across the encoder and decoder was developed, and has proved to be effective at perceptual defect identification with high pixel-wise accuracy across all three prototypical defect classes. Direct comparison between the *DefectSegNet* prediction and ground truth using color-coded confusion matrices revealed that uncommon feature representation, particularly those with divergent contrast, is one of the main sources of uncertainties in model prediction. This, in turn, confirmed that the prior efforts on improving input defect image quality have not only led to a ground truth with high fidelity, but also promote feature homogeneity in training data and thus advance model performance. Moreover, we found that the training data is better assessed by also taking feature density and consistency into consideration for pixelwise semantic segmentation tasks.

The application of the *DefectSegNet* predicted defect maps to quantifying materials metrics, in general, outperformed the manual quantification by human experts. This is particularly advantageous for the analysis of high-density features, which are critical for understanding extreme processing/degradation conditions, but is time demanding and error-prone in conventional manual counting. We conclude that the deep learning semantic segmentation established on advanced microscopy and on optimized CNN architecture offers a path forward to the high-throughput defects quantification needed for rational alloy design.

## Supplementary information

SI

## Data Availability

The datasets generated during and/or analyzed during the current study are available from the corresponding author on reasonable request.
