# Supplementary material for: Deep Learning for Semantic Segmentation of Defects in Advanced STEM Images of Steels
Source: Sci Rep. 2019 Sep 4;9:12744. doi: 10.1038/s41598-019-49105-0 (PMC6726638; doi:10.1038/s41598-019-49105-0)
Supplement: Supplementary file 1 — SI [file 41598_2019_49105_MOESM1_ESM.docx]

**Supplementary information**

**Deep Learning for Semantic Segmentation of Defects in Advanced STEM Images of Steels**

**Graham Roberts^1^, Simon Y. Haile^2^, Rajat Sainju^3^, Danny J. Edwards^1^, Brian Hutchinson^2,4^ and Yuanyuan Zhu^1,3*^**

1Nuclear Sciences Division, 4Computing and Analytics Division, Pacific Northwest National Laboratory, Richland, WA 99352, USA

2Computer Science Department, Western Washington University, Bellingham, WA 98225, USA

3Department of Materials Science and Engineering, Institute of Materials Science, University of Connecticut, Storrs, CT 06269, USA
*yuanyuan.2.zhu@uconn.edu

**
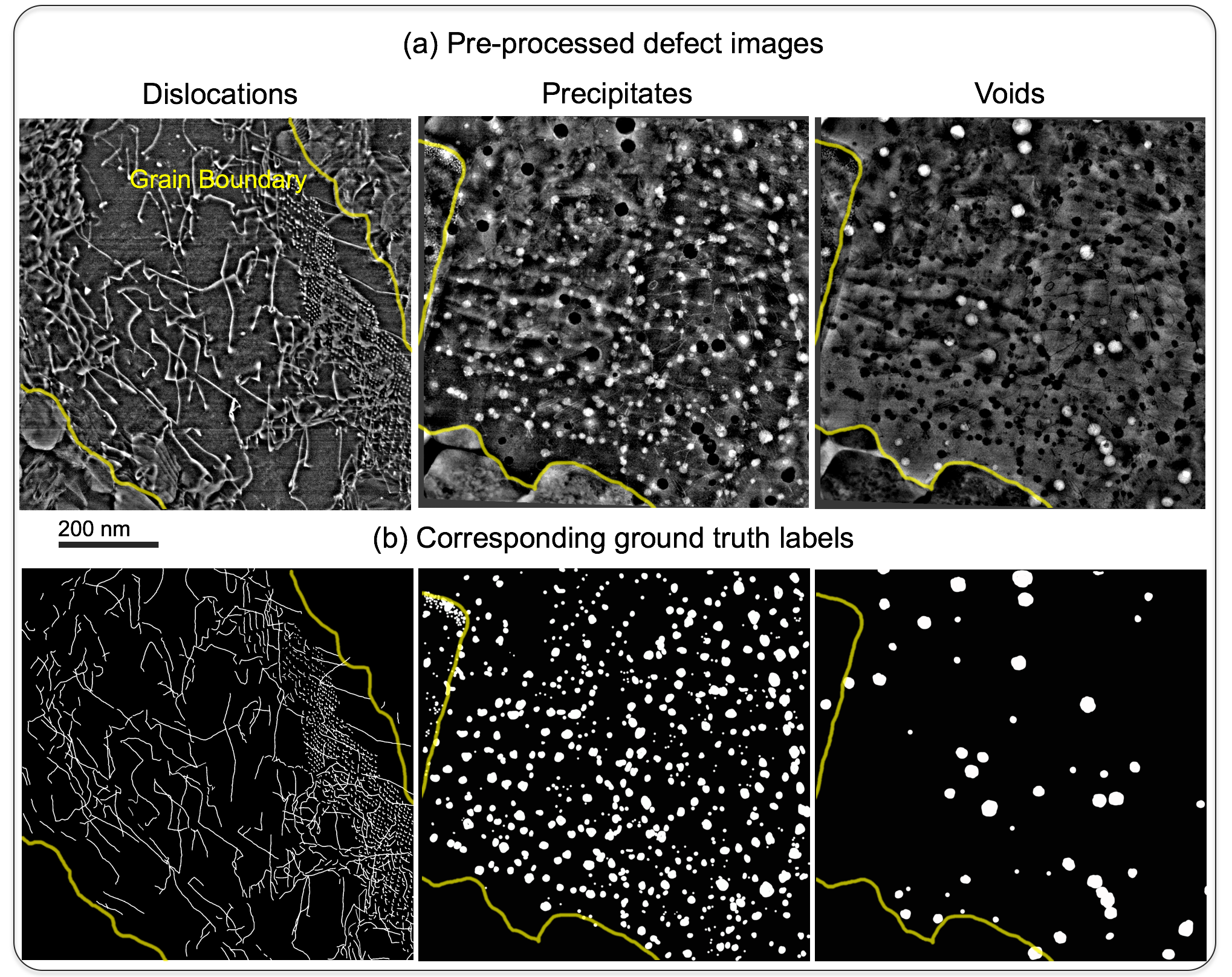
**

**Figure S1.** An example overview of pre-processed defect images and ground truth labels of the three crystallographic defect classes. Grain boundaries were manually identified for the calculation of defect quantification metrics.

| Architectures | **Pixel accuracy** | | | | **IU** | | | | |
| --- | --- | --- | --- | --- | --- | --- | --- | --- | --- |
|  | Dislocations | Precipitates | Voids | **Overall defect** | | Dislocations | Precipitates | Voids | **Overall defect** |
| **Unet-VGG (19)** | 87.01±1.6% | 93.2±0.78% | 98.84±0.7% | 89.49±1.03% | | 32.96±4.38% | 58.68±1.03% | 78.43±4.75% | 56.69±3.39% |
| **Deep Unet (26)** | 90.35±1.29% | 83.16±3.15% | 92.37±1.57% | 88.63±0.7% | | 40.01±1.56% | 30.56±5.29% | 12.36±5.36% | 27.64±4.07% |
| **DenseNet-VGG (19)** | 91.1±1.4% | 93.37±1.49% | 98.68±0.67% | 94.38±1.19% | | 42.04±2.36% | 59.83±4.07% | **81.25±5.68%** | 61.04±4.04% |
| **Enhanced DenseNet-VGG (19), the *DefectSegNet* in this work** | **91.6±1.77%** | **93.38±1.07%** | **98.85±0.56%** | **94.61±1.13%** | | **44.34±0.63%** | **59.85±2.07%** | 81.19±3.68% | **61.79±2.13%** |

Table S1. Results from *DefectSegNet* are compared to results obtained using three other DCNN architectures for pixel-wise sematic segmentation of the three defect features, presenting the best IUs for dislocations and the overall defects. Here, Unet-VGG is an architecture inspired by 19-layer VGG^1^ incorporate with the U-Net^2^, Deep Unet is similar to the 26-layer U-Net^2^, DenseNet-VGG is based on the Unet-VGG with dense blocks as introduced inDenseNet^3^, *DefectSegNet* is similar to DenseNet-VGG but with dense skip connections as shown in the Fig. 4 in the main text. Note that the results above are not an exhaustive comparison of the design in deep learning architecture and its resulting performance. To accomplish this, a large and feature diverse training date set need to be included to fully evaluate the effects of architecture. Nevertheless, the present training results, in particular for the dislocations, show an improvement in IU performance when the *DefectSegNet* was adopted.

References

1. Simonyan, K. & Zisserman, A. Very deep convolutional networks for large-scale image recognition. *In: ICLR*, (2015).

2. Ronneberger, O., Fischer, P. & Brox, T. 234-241 (Springer International Publishing).

3. Huang, G., Liu, Z., Maaten, L. v. d. & Weinberger, K. Q. Densely Connected Convolutional Networks. *IEEE Conference on Computer Vision and Pattern Recognition (CVPR)*, (2017).
